# Cutaneous epithelioid angiomatous nodule: a report of a series including a case with moderate cytologic atypia and immunosuppression

**DOI:** 10.1186/s13000-018-0729-5

**Published:** 2018-08-13

**Authors:** Runjan Chetty, Zaid S. Kamil, Ami Wang, Ayman Al Habeeb, Danny Ghazarian

**Affiliations:** Department of Pathology, Laboratory Medicine Program, University Health Network and University of Toronto, 11th floor Eaton wing, Toronto General Hospital 200 Elizabeth Street, Toronto, M5G 2C4 Canada

**Keywords:** Cutaneous, Vascular tumour, Epithelioid, Immunosuppression, Angiomatous nodule

## Abstract

**Background:**

Cutaneous epithelioid angiomatous nodule (CEAN) is a very rare and relatively recently recognized vascular proliferation characterized usually by minimal cytological atypia and accompanying mitotic activity. As such, CEAN represents an important diagnostic pitfall, which could lead to significant misdiagnosis and unnecessary treatment.

**Methods:**

The clinicopathologic findings of 5 cases of CEAN were reviewed including a unique case with typical findings but also moderate cytologic atypia and brisk mitotic activity in a patient on immunosuppression.

**Results:**

The cases were in 3 women and 2 men ranging in age from 18 to 61 years with lesions in the neck (2 cases), upper arm, back and shoulder. In 4 of the cases, the patients did not have any relevant potentially contributory clinical history, and in 1 case the patient was on immunosuppressive treatment. All 5 cases were superficially located within the dermis, well-circumscribed and similarly composed of epithelioid cells displaying minimal (in 4 cases) and moderate (1 case) atypia. The mitotic count ranged from 1 to 3 per 10 high power fields (HPF) in 4 cases and up to 9 per 10 HPF in the immunosuppressed patient. Atypical mitoses were not encountered in any of the cases. Two lesions that were incompletely excised recurred, but none of the patients showed distant metastases.

**Conclusion:**

While cytologically alarming, CEAN has a characteristic microscopic appearance and if completely excised follows an indolent course.

This work was presented at the United States and Canadian Academy of Pathology 104th Annual meeting [1].

## Background

Cutaneous epithelioid angiomatous nodule (CEAN) is a rare and relatively recently recognized vascular lesion. This benign entity can be confused easily with benign and malignant vascular tumours such as pyogenic granuloma, epithelioid haemangioma, and bacillary angiomatosis, Kaposi’s sarcoma (especially when arising in immunocompromised patients), epithelioid hemangioendothelioma and angiosarcoma. In all cases reported so far, minimal to mild cytological atypia and absence of invasion has been described as reliable features to distinguish CEAN from malignant vascular tumours. In addition to 4 cases displaying mild cytological atypia, we report an unusual case of CEAN with moderate cytologic atypia and brisk mitotic activity in an immunosuppressed patient. We also present the defining clinical and pathological features that would enable pathologists to correctly classify these unusual epithelioid vascular lesions.

## Methods

Five cases of CEAN, including 4 in-house cases and 1 case received in consultation, were retrospectively identified in the Pathology database at the University Health Network, Toronto, Ontario, Canada between 2012 and 2014. Where available, the clinical features were reviewed using electronic patient records. The pathological characteristics of the 5 cases were reviewed including hematoxylin and eosin and immunohistochemical stained sections. In terms of assessing atypia in these lesions, the lesional cells were compared to normal endothelial cells in terms of lesional cell size and nuclear features such as size, hyperchromasia and presence of nucleoli. The comparison was graded as mild or moderate depending on similarity to normal non-lesional endothelial cells.

A review of the pertinent literature was also performed.

## Results

The cases were in 3 females and 2 males with an age range from 18 to 61 years (mean 38 years).

Clinically, all the lesions presented as well-circumscribed, solitary erythematous papules or nodules on the head and neck (2 cases), shoulder, arm and back regions. One patient (case 5) was on immunosuppression therapy. Two cases were incompletely excised and presented with recurrent lesions. Case 1 had 3 lesions closely clustered on the arm.

**Fig. 1:**
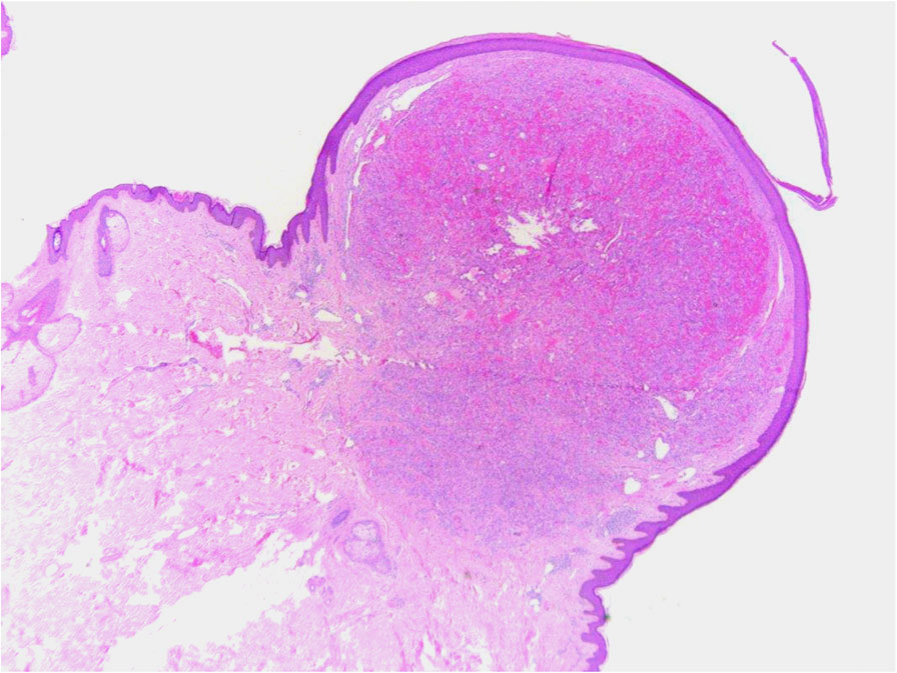
CEAN from case 1 showing a typically polypoid lesion with a well-circumscribed vascular dermal nodule. There is a suggestion of a collarette at the edges of the lesion simulating pyogenic granuloma

**Fig. 2:**
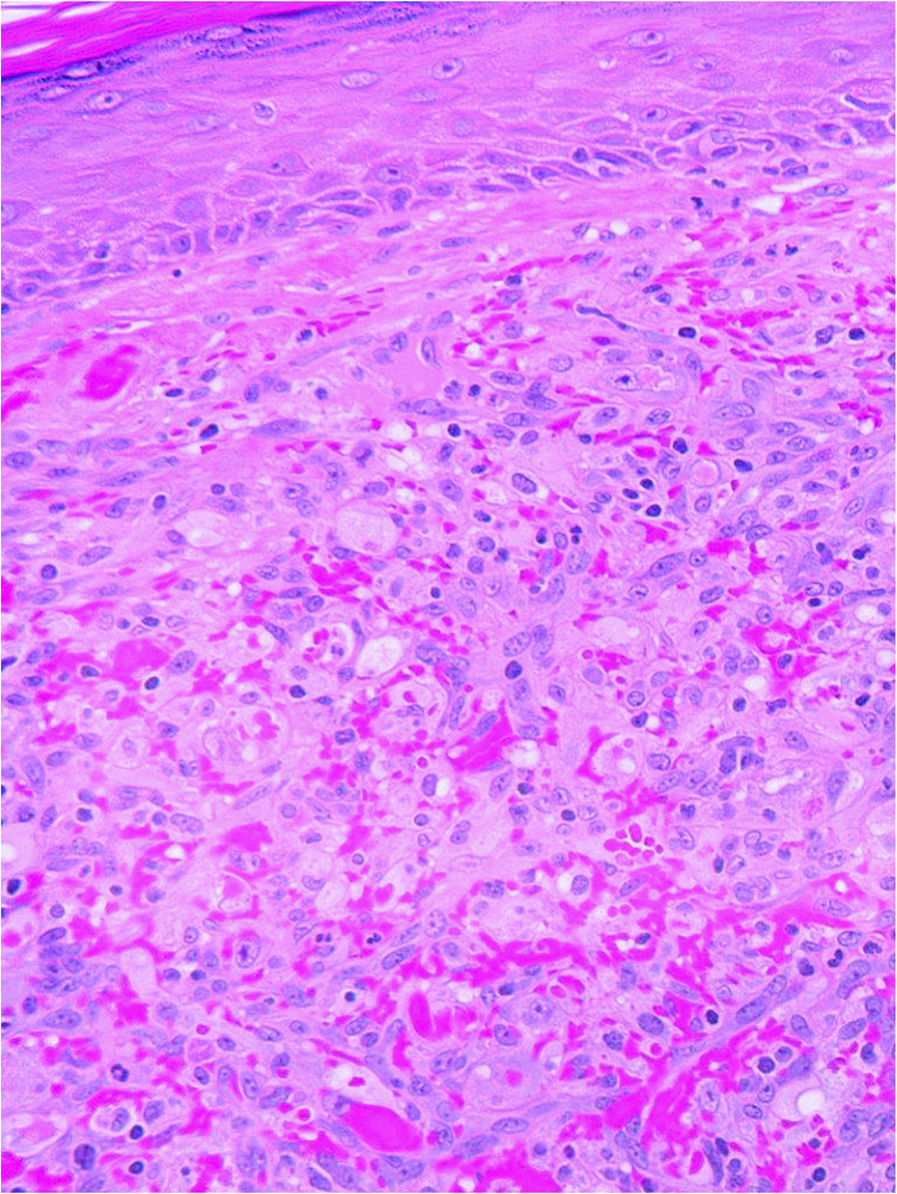
Case 3 showing epithelioid cells exhibiting mild cytological atypia, large nuclei with easily discerned nucleoli, cytoplasmic vacuoles, occasional mitoses and an inflammatory infiltrate of lymphocytes, plasma cells and occasional eosinophils

Histologically, the 4 typical cases of CEAN were characterized by polypoid, well-circumscribed, dermal (Fig. 1), non-encapsulated, unilobular solid proliferation of predominantly epithelioid cells and very occasional spindled cells with vesicular nuclei, conspicuous nucleoli, abundant eosinophilic to clear cytoplasm and occasional intracytoplasmic vacuoles. Vascular channels lined by similar cells were also part of the lesions. Within the stroma the inflammatory cell component consisted of lymphocytes, plasma cells and occasional eosinophils. The cytological atypia in these cases ranged from mild in 4 cases and moderate in 1 case (Fig. 2). Mitotic activity ranged from 3 to 9/10 HPF. One of these 4 cases (case 1) recurred after incomplete excision.

**Fig. 3:**
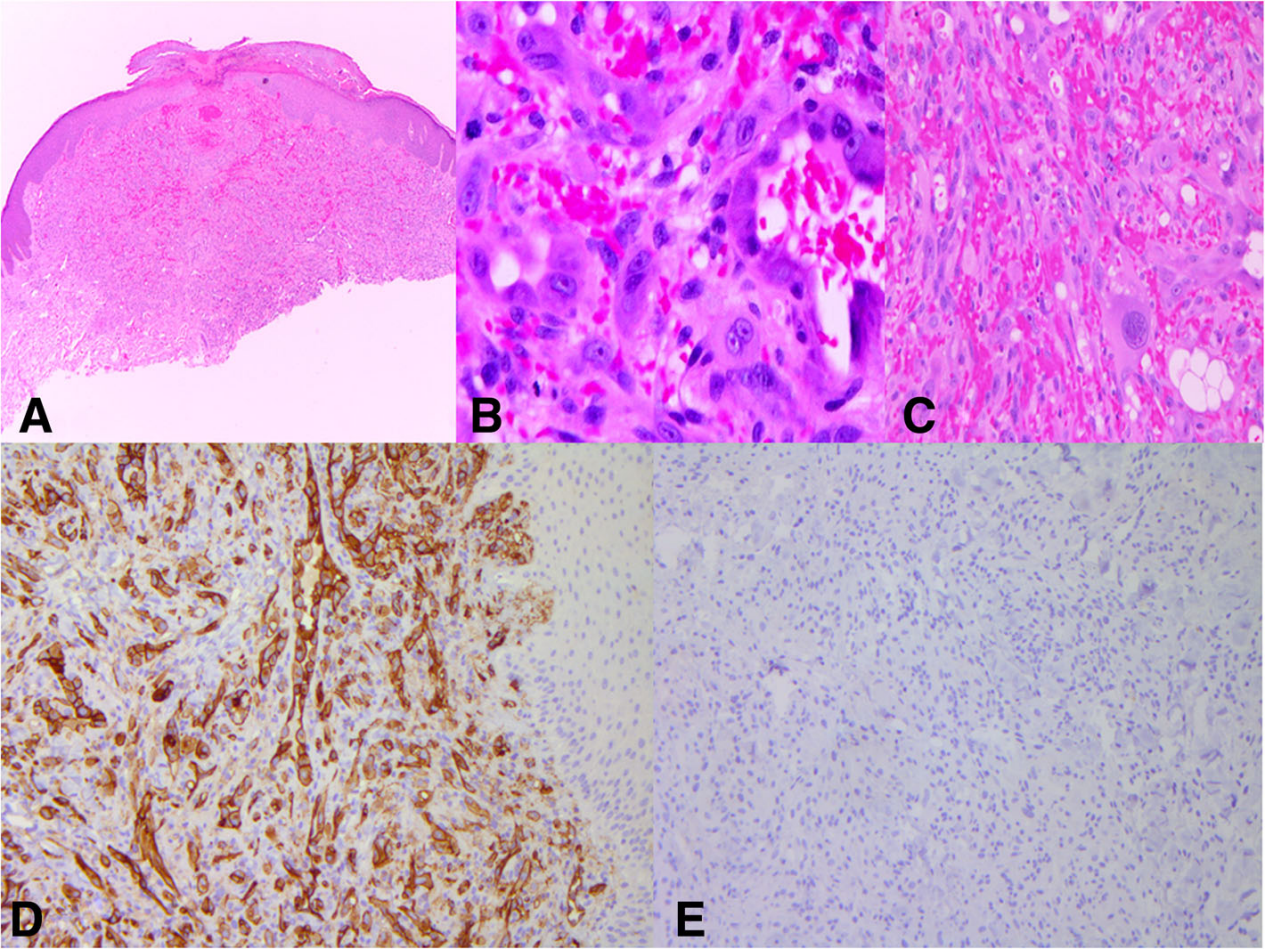
**a-e** Case 5, from the initial lesion that recurred, showing a reasonably well-circumscribed and delineated lesion that is incompletely excised (**a**). Higher magnification of the lesion shows a dermal proliferation of moderately atypical epithelioid cells. Several cells have cytoplasmic vacuoles (“blister cells”) (**b**). There is a s dermal proliferation of moderately atypical epithelioid cells including a giant mononuclear cell (**c**). CD31 immunopositivity highlighting the vascularity of CEAN in case 5 (**d**); in addition, HHV8 is negative (**e**)

The unusual case (case 5) which was incompletely excised and presented with a recurrence, had a well circumscribed appearance (Fig. 3a**)** with moderate cytologic atypia (Fig. 3b), including occasional giant cells (Fig. 3c) and mitotic activity up to 9 per 10 HPF was noted in both the primary and recurrent lesions. The recurrent lesions in the 2 cases were histologically similar to the original CEAN.

In all 5 cases, there was no evidence of sheet-like solid growth pattern, infiltrative margins, atypical mitoses or necrosis. Metastases were not recorded in any of the cases for the duration of follow-up ranging from 3 to 24 months after diagnosis.

By immunohistochemistry, the lesional cells were variably positive for vascular markers CD31 (Fig. 3d) and CD34 and are negative for Human herpes virus-8 (HHV-8) (Fig. 3e) and cytomegalovirus (CMV). Warthin-Starry stains and EBER in-situ hybridization were negative.

## Discussion

Cutaneous epithelioid angiomatous nodule is a rare, benign, reactive vascular proliferation that was first described by Brenn and Fletcher in 2004 [2]. Since then, there have been in excess of 20 case reports and or small series in the English literature [2–22]. Clinically, these lesions present as a rapidly growing small, solitary nodules or papules, however; multiple lesions have also been reported [2–4, 8, 12, 14, 15]. The trunk and extremities are the most common site of occurrence with no particular gender predilection. Other reported sites include: head and neck and vulvar region. Histologically, the lesions are non-capsulated, well-circumscribed, unilobular superficial dermal nodules with intact or ulcerated overlying epidermis. They are composed of solid proliferation of plump epithelioid cells, as well as thin-walled vascular channels lined by similar cells that have eosinophilic cytoplasm, vesicular nuclei and prominent nucleoli [2, 3]. Mitotic activity is usually present; however, atypical mitotic forms and necrosis are not encountered. Cases with “abundant” mitotic activity have been described [3, 13]. Unfortunately, the actual mitotic count in these cases was not provided. Immunohistochemistry shows the lesional cells to be immunoreactive for vascular markers (CD34, CD31, factor VIII-related antigen, D2–40) and focally for SMA, and all lesions are negative for HHV-8. Simple complete excision is curative [2].

We report 5 patients with CEAN. All lesions were circumscribed, dermal nodules composed of plump epithelioid cells without necrosis or atypical mitoses and no extension into the subcutaneous tissue. The degree of cytological atypia in 4 cases ranged from mild to moderate in concordance with the previous reported cases. Interestingly though; case 5 was characterized by a moderate cytological atypia with pleomorphic nuclei and mitotic count of up to 9 mitosis/10 HPFs. The moderate cytological atypia in case 5, which was more pronounced in the recurrent lesion may be attributed to immunosuppression. A background of immunosuppression has not been reported in the literature previously in CEAN and this unusual case represents the first report in association with immunosuppression. It should be noted that other than the increased cytological atypia and higher mitotic rate, the lesion otherwise fulfilled the morphological criteria for a CEAN. Case 1 presented with multiple lesions; typically CEAN is solitary lesion but multiple eruptive clusters have been documented by Sangueza and colleagues [4]. We would recommend complete excision of all lesions if multiple lesions are encountered. One of the three lesions in case 1 was incompletely excised and recurred after 9 months.

CEAN should be differentiated from other vascular lesions [2–4, 8, 9, 13, 16, 23]. Pyogenic granuloma can be distinguished from CEAN as it usually presents on the face as a multilobulated lesion following trauma. Trauma has not been reported to date in cases of CEAN. Bacillary angiomatosis is characteristically seen in immunosuppressed patients, shows a deep neutrophilic infiltrate, vasoformative architecture, and aggregates of eosinophilic granular material (that are positive on a Warthin Starry stain) representing clusters of *Bartonella henselae* organisms. In both pyogenic granuloma and bacillary angiomatosis, the vessels are not usually lined by epithelioid appearing endothelial cells. However, rare cases of pyogenic granuloma with focal epithelioid cells have been described but a pure epithelioid pyogenic granuloma is not thought to exist [2]. Separation from CEAN is therefore, based on the presence of a very exophytic, polypoid appearance and absence of epithelioid cells lining vascular channels in pyogenic granuloma. Epithelioid hemangioma can be differentiated from CEAN by its predominant vasoformative architecture, extension into the subcutis, multilobulation, involvement of small muscular vessels, and presence of lymphoid follicles [8, 16]. Epithelioid hemangioendothelioma is an infiltrative malignant neoplasm, often arises in solid organs and deep soft tissue, and arranged in cords and strands embedded in myxohyaline stroma and have the characteristic blister cells.

Most importantly, CEAN should not be confused with angiosarcoma, especially those cases of CEAN presenting with moderate cytologic atypia and more than 5 mitoses/10 HPF as in case 5 described herein in association with immunosuppression. Angiosarcomas are usually larger, show solid sheet-like growth widely infiltrative borders, dermal collagen dissection by vascular channels, necrosis, atypical mitoses and can obviously present with metastases. CEAN that belong to the cytologically blander end of the spectrum can be more easily separated from a malignant vascular neoplasm. The greatest difficulty is encountered in CEAN cases that have moderate cytologic atypia as seen in case 5 of this series. However, in keeping with CEAN rather than angiosarcoma, this case did not metastasize although local recurrence occurred because of incomplete initial excision. Histologically, the lesion maintained circumscription, was not infiltrative or destructive locally, and did not harbor atypical mitoses or necrosis. Truly malignant vascular neoplasms are aggressive biologically and show the microscopic features of this inherent biology.

The general consensus is that CEAN is closely related to epithelioid hemangioma/pyogenic granuloma and sometimes the distinction between these entities can be very challenging. Of interest, recent report about frequent *FOS* gene rearrangements in cases of atypical epithelioid hemangiomas has been described and whether such rearrangements can be detected in cases of CEAN have yet to be investigated [24, 25].

## Conclusions

CEAN is a rare benign epithelioid vascular proliferation that can be associated with immunosuppression and shows cytologic atypia and brisk mitotic activity, features that can be mistaken for malignant vascular tumors. However, CEAN does not show other malignant features such as atypical mitoses, necrosis, an infiltrative growth pattern or metastases. We emphasize the relationship of the atypical features with the patient’s impaired immune status in one of our cases which should be explored in future studies to avoid the faulty diagnosis of more ominous lesions.

## Abbreviations

CEAN: Cutaneous epithelioid angiomatous nodule
CMV: Cytomegalovirus
HHV-8: Human herpes virus-8
